# Acute Exacerbation of Rheumatoid Arthritis Misdiagnosed as COVID-19: A Case Report

**DOI:** 10.3389/fmed.2022.844609

**Published:** 2022-03-10

**Authors:** Serife Torun, Irem Karaman

**Affiliations:** ^1^Department of Pulmonary Diseases, Konya Training and Research Hospital, Konya, Turkey; ^2^School of Medicine, Bahcesehir University, Istanbul, Turkey

**Keywords:** rheumatoid arthritis, interstitial lung disease, COVID-19, diagnosis, misdiagnosis

## Abstract

**Background:**

Rheumatoid arthritis (RA) is a systemic inflammatory connective tissue disease that affects 1–2% of the population worldwide. Pulmonary manifestations including interstitial lung disease (ILD), airway disease, pleural and vascular disease can be seen in up to 30–40% of patients with RA, which are recognized as the second most frequent cause of death in RA patients. The simultaneous occurrence of COVID-19 in RA patients with or without ILD, and the similarities and differences between RA-related ILD and COVID-19 lung findings have been reported in the literature. However, there was no reported case on differentiation of clinical findings of a patient with RA exacerbation causing a new diagnosis of ILD during the pandemic conditions.

**Case Presentation:**

Here, we presented a patient with RA who was misdiagnosed as COVID-19 twice due to non-specific respiratory symptoms and ground-glass opacities observed in high-resolution CT. The misdiagnosis led to a delayed diagnosis of ILD and prolonged pulmonary symptoms.

**Discussion:**

Clinicians must critically review patients throughout the diagnostic workup by thinking other diseases besides COVID-19, particularly in the absence of a confirmatory result. The link between ILD or ILD exacerbation and COVID-19 remains to be determined. While research continues in the field, it is important to consider the importance of COVID-19 in cases of ILD exacerbation, and vice versa.

**Conclusion:**

Distinguishing lung imaging findings of COVID-19 from ILD is a major concern. Even though the primary manifestation of COVID-19 consists of respiratory symptoms, clinicians should be vigilant for other common conditions having the same symptoms. Clinicians should carefully distinguish a differential diagnosis between COVID-19 and a flare of rheumatic disease.

## Introduction

Rheumatoid arthritis (RA) is a systemic inflammatory connective tissue disease (CTD) which affects 1–2% of the population worldwide (1). Although RA causes symmetrical, progressive and erosive arthritis that mostly involves small joints, extra-articular manifestations of RA are common including skin, eye, heart lung, renal, nervous and gastrointestinal systems, which are mostly seen in patients with active and severe RA, with RF and/or HLA-DR4 positivity (1). Systemic manifestations include visceral nodules, vasculitis, Sjogren’s syndrome, and pulmonary fibrosis (1).

Pulmonary manifestations can be seen in up to 30–40% of patients, which are recognized as the second most frequent cause of death in RA patients, after cardiovascular disease (2). Pulmonary manifestations include interstitial lung disease (e.g., usual interstitial pneumonia, non-specific interstitial pneumonia, organizing pneumonia, lymphocytic interstitial pneumonitis, mixed lung disease), rheumatoid nodules, airways disease (e.g., bronchiectasis, bronchiolitis, bronchiolitis obliterans, follicular bronchiolitis, panbronchiolitis, chronic small airway obstruction, cricoarytenoid arthritis), pleural disease (pleural effusion, pleuritis, pleural thickening, lung entrapment and trapped lung, and pneumothorax), vascular disease (e.g., pulmonary hypertension, vasculitis, haemorrhagic alveolitis, venous thromboembolism) and diffuse alveolar hemorrhage. RA involvement of lungs can also cause other comorbidities such as Caplan syndrome (rheumatoid pneumoconiosis), amyloidosis, lung cancer, and drug-induced pulmonary adverse events (2).

Interstitial lung disease (ILD) is a life-threatening extra-articular manifestation of rheumatoid arthritis (RA). Differential diagnosis of different forms of rheumatoid arthritis related interstitial lung disease can be made radiologically, which depends on the pattern of distribution (peripheral, subpleural, basal, peribronchial, diffuse or focal, predominantly in the upper or lower, and symmetric), or findings (reticular opacities, honeycombing, minimal or extensive ground- glass opacity, architectural distortion, irregular linear opacities, traction bronchiectasis, subpleural preservation, consolidations; reversed halo sign, centrilobular nodules, and thin-walled cysts). Ground-glass opacities and consolidations are the most common findings which are seen in almost all interstitial lung diseases caused by RA (3).

Severe acute respiratory syndrome coronavirus 2 (SARS-CoV-2), the novel virus that causes coronavirus disease 2019 (COVID-19) pandemic, primarily causes a respiratory illness that is mostly characterized by widespread ground glass opacities and consolidations in the lungs (4). The radiological and clinical similarities between other interstitial lung diseases and COVID-19, together with the pandemic circumstances, may cause many false misdiagnoses of COVID-19, particularly since clinicians are faced with a new and evolving disease that can cause confusion and increase the risk of mistakes (4, 5).

The simultaneous occurrence of COVID-19 in RA patients with or without ILD, and the similarities and differences between RA-related ILD and COVID-19 lung findings have been reported in the literature (4–7). However, there was no reported case on differentiation of clinical findings of a patient with RA exacerbation causing a new diagnosis of ILD during the pandemic conditions. Here, we presented a case with ILD-related RA, which was misdiagnosed as COVID-19 and caused a delayed diagnosis.

## Case Presentation

**FIGURE 1:**
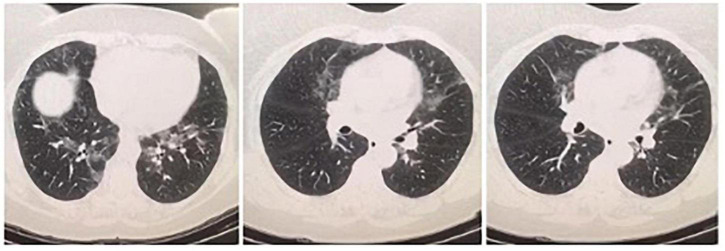
On her first HRCT result, bilateral central and peripheric ground glass opacities in upper and lower lung lobes and nodular thickening of right posterior pleura were noticed.

**FIGURE 2:**
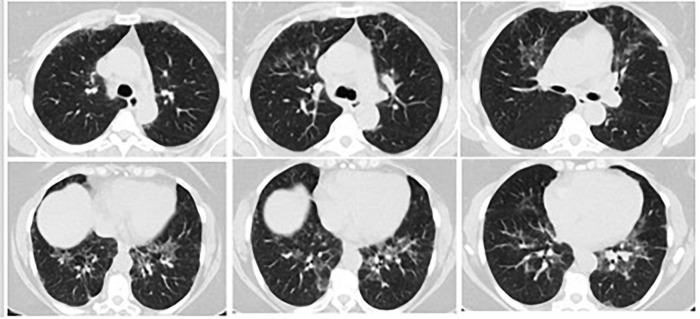
Three months later, on her repeated HRCT, bilateral peripheral peribronchovascular fibrotic reticulations in upper and lower lung lobes and increased areas of ground-glass opacities with pleural thickening around right posterior lung lobe were observed.

A 59-year-old female patient was consulted to the chest diseases outpatient clinic with complaints of dyspnea, deteriorated general condition and general articular pain. She stated that her complaints had been continuing for a year and increased in the last 3 months. In the weeks prior, the patient noted malaise, subjective fevers, and upper respiratory symptoms including dry cough and sore throat. Three months before, she was diagnosed and treated with a COVID-19 diagnosis due to HRCT findings resembling COVID-19 in the clinic she was applied to due to same complaints (Figure 1). Her real-time polymerase chain reaction (PCR) result for SARS-CoV-2 at that time was negative. Three months later, she again referred to clinics due to persistent upper respiratory complaints and HRCT findings (Figure 2) and received a course of COVID-19 treatment with favipiravir. She had two PCR results within 3 months for SARS-CoV-2 which was both negative.

She consulted to our clinic for further evaluation since her symptoms did not improve. On admission, the patient had mild respiratory symptoms, and her vital signs were as follows: blood pressure, 129/79 mmHg; heart rate, 79 beats/min; body temperature, 37.0°C; and respiratory rate, 18 breaths/min. On physical examination of the patient, deformations and rheumatoid nodules in the fingers were seen. Other systemic examination findings were unremarkable. When the medical history of the patient was questioned, she reported that she had been diagnosed with RA for 13 years, and she was using her prescribed medications hydroxychloroquine, sulfasalazine, and occasionally prednisolone. However, she reported that she was not going to her doctor appointments during the last year due to COVID-19 pandemic and she discontinued all her medications. She did not have any other comorbidities. The patient denied any smoking and alcohol drinking habits.

On her first HRCT result, bilateral central and peripheric ground glass opacities in upper and lower lung lobes and nodular thickening of right posterior pleura were noticed (Figure 1). Three months later, on her repeated HRCT, bilateral peripheral peribronchovascular fibrotic reticulations in upper and lower lung lobes and increased areas of ground-glass opacities with pleural thickening around right posterior lung lobe were observed (Figure 2). Her blood test results at the time of admission were unremarkable except CRP: 14.2 mg/L. Her microbiological test results for hepatitis B, hepatitis C, and Brucella were negative. Her SARS-CoV-2 IgM and IgG results were both negative.

After a careful evaluation of patient’s history and clinical findings, a new diagnosis of ILD related to RA was made. She was prescribed with cyclophosphamide and discharged for routine follow up.

## Discussion

In December 2019, an outbreak of pneumonia caused by a novel coronavirus occurred in Wuhan, China and affected the whole world since then. Herein, we reported a patient with RA who was misdiagnosed as COVID-19 twice due to non-specific respiratory symptoms and ground-glass opacities observed in high-resolution CT. The misdiagnosis led to a delayed diagnosis of ILD and prolonged pulmonary symptoms. Even though the primary manifestation of COVID-19 consists of respiratory symptoms, clinicians should be vigilant for other common conditions having the same symptoms (8). During the pandemic, clinicians will be expected to adapt by incorporating COVID-19 into their routine clinical practices. Clinicians must critically review patients throughout the diagnostic workup by thinking other diseases besides COVID-19, particularly in the absence of a confirmatory result. Although the negative predictive value of COVID-19 (up to 99.0%) is high, the positive predictive value of a finding for COVID-19 ranges from 1.5 to 8.3%; hence, a positive chest CT test result should not warrant a diagnostic closure (9). In addition, although negative PCR results cannot exclude the diagnosis of COVID-19, it should direct clinicians to think for other possible entities by considering a through medical history.

Interstitial lung disease (ILD) is a heterogeneous group of disorders characterized by dyspnea and bilateral infiltrations of the lung. While no cause is found in idiopathic cases (e.g., idiopathic pulmonary fibrosis), other causes include systemic illnesses (e.g., autoimmune disorders), environmental exposures (e.g., asbestosis, hypersensitivity pneumonitis), or drug-induced (5). Although ILD is increasingly being recognized as a manifestation of RA, one third of patients are believed to have subclinical disease, which delays diagnosis and intervention. Since viral infection has been implicated as an important cause of ILD exacerbation, recent concerns have been raised by the potential impact of the COVID-19 pandemic on ILD (5). In individuals with underlying ILD, COVID-19 has prompted a number of challenges such as diagnostic uncertainty and uncertainties about the impact of immunosuppressive agents (5). However, our case of an acute exacerbation of ILD in a RA patient during the COVID-19 pandemic raises a number of important concepts, such as whether or not if the development of RA was triggered by COVID-19, if not, whether or not COVID-19 pandemic caused a misleading delayed diagnosis. The negative results of SARS-CoV-2 PCR and SARS-CoV-2 IgM and IgG suggest that the patient was not infected with SARS-CoV-2, however, her condition of RA was deteriorated, and her diagnosis of ILD was delayed due to COVID-19 pandemic. In our patient, ILD exacerbation was most likely due to the patient’s failure to comply with her RA treatment. It is also possible that acute exacerbations of ILD could have occurred during the pandemic due to the setbacks in the follow-up of chronic diseases. Therefore, the link between ILD or ILD exacerbation and COVID-19 remains to be determined. While research continues in the field, it is important to consider the importance of COVID-19 in cases of ILD exacerbation, and vice versa.

Inadequate history-taking and physical examination at the time of initial presentation are the most common reasons causing a delay in diagnosis by a premature diagnostic closure related to COVID-19. A diagnostic error is the failure to establish an accurate or timely diagnosis. These diagnostic errors can either be caused by cognitive errors (such as symptomatology, and misdirected investigations) or system-related errors (such as lack of standardization in the orientation provided to front-line workers during the pandemic with a consistently evolving protocols and guidelines regarding COVID-19) (10). Furthermore, clinicians’ initial diagnostic assessment was likely limited by the fear of infection by SARS-CoV-2 which led clinicians to spend less time on the history and physical examination than they normally would have. Although the vigilance and precautions needed for patients suspected of having COVID-19 should not be relaxed, fear should not preclude us from delivering appropriate care. Unfortunately, the hesitation of patients to go to the hospital to avoid exposure in COVID-19 era and the limitation of resources have created significant barriers for evaluation (8). Even in the case of patients who are already admitted to the clinics, when the suspicion of COVID-19 is raised, it surely affects care by distracting providers from other diagnoses.

In conclusion, we would like to emphasize the importance of the evaluation of the complete history and previous and follow-up imaging of patients regarding a possible co-existing condition that may cause pulmonary involvement during COVID-19 pandemic. Clinicians should carefully distinguish the differential diagnosis of a flare of rheumatic disease from COVID-19 by proceeding with further testing, particularly in the presence of negative PCR results.

## Data Availability Statement

The original contributions presented in the study are included in the article/supplementary material, further inquiries can be directed to the corresponding author.

## Ethics Statement

Ethical review and approval was not required for the study on human participants in accordance with the local legislation and institutional requirements. The patients/participants provided their written informed consent to participate in this study.

## Author Contributions

Both authors have contributed sufficiently in the design of the study, preparation of the manuscript, read, and approved the final version of the manuscript.

## Conflict of Interest

The authors declare that the research was conducted in the absence of any commercial or financial relationships that could be construed as a potential conflict of interest.

## Publisher’s Note

All claims expressed in this article are solely those of the authors and do not necessarily represent those of their affiliated organizations, or those of the publisher, the editors and the reviewers. Any product that may be evaluated in this article, or claim that may be made by its manufacturer, is not guaranteed or endorsed by the publisher.
